# Skin-sparing mastectomy and radiotherapy: an update

**DOI:** 10.1186/1477-7800-3-35

**Published:** 2006-10-17

**Authors:** Ramia Mokbel, Kefah Mokbel

**Affiliations:** 1 St. George's & The Princess Grace Hospitals, London, UK

## Abstract

Despite the lack of randomised controlled trials and paucity of the published data, the current evidence suggests that the post-mastectomy radiation therapy (PMRT) does not represent a contraindication to skin-sparing mastectomy (SSM) and immediate breast reconstruction (IBR) in the multidisciplinary setting. Although PMRT is associated with a higher incidence of complications, a satisfactory cosmetic outcome can be achieved in most patients. Radiation has a deleterious effect on autologous flap reconstruction that relies on fat for volume replacement such as the deep inferior epi-gastric perforator (DIEP) flap reconstruction and this method of reconstruction should be delayed until RT is completed. Until better methods of RT delivery are developed to minimise complications, women at high risk of requiring PMRT, can be safely offered SSM and IBR with a sub-pectoral saline-filled tissue expander and this can be replaced with a permanent prosthesis or converted into an autologous flap reconstruction after the completion of RT. Any capsule formation can be surgically treated at this stage. This new concept, known as immediate-delayed reconstruction, can avoid the cosmetic and RT delivery problems that can occur after IBR.

Furthermore, prior RT does not represent a contra-indication to SSM and IBR, however it increases the incidence of complications.

## Background

Both immediate and delayed breast reconstruction following mastectomy provide substantial psychosocial benefits for women undergoing mastectomy for breast cancer [1]. Despite the lack of randomised controlled trials, skin-sparing mastectomy (SSM) has become an accepted procedure in women undergoing mastectomy and immediate reconstruction for early breast cancer. Conventional non-skin sparing mastectomy (NSSM) often results in prominent scars on the new breast and a paddle of skin that is of a different colour and texture. SSM preserves most of the overlying skin during immediate breast reconstruction (IBR) thus leading to a superior aesthetic outcome. It also reduces the need for contralateral breast adjustment in order to achieve symmetry [2]. Compared to non-skin-sparing mastectomy (NSSM), SSM seems to be oncologically safe in patients undergoing mastectomy for stage I breast cancer, ductal carcinoma in situ (DCIS), or risk-reduction [2]. In the absence of extensive skin involvement by tumour, multifocality, T2 tumours and nodal positivity do not represent contra-indications to SSM in relation to oncological safety [2], however its role in the treatment of stage II or III breast cancer is debatable in view of the high probability of requiring post-mastectomy radiotherapy (RT). In addition to discussing RT after SSM, this article explores the feasibility of SSM after prior RT.

## RT after SSM

Most women undergoing mastectomy for breast cancer do not require post-operative RT. However, patients with several positive regional lymph nodes and/or large tumours are offered RT in view of the proven reduction in loco-regional recurrence and improved survival [3]. Similarly, RT is also indicated in some patients who have undergone SSM and IBR. Recently, the incidence of post-mastectomy RT has been increasing thus raising concerns regarding its effects on the reconstructed breast and the potential interference of IBR with RT delivery [4,5]. There is a lack of prospective trials concerning the use of RT with SSM and most of the published evidence is derived by enthusiasts from single-centres and NSSM cases.

Although results from individual series vary considerably, it appears that the complications of RT following IBR occur in a high proportion of patients [6].

A study of immediate TRAM reconstructions showed the commonest complications were fat necrosis (16%) and radiation fibrosis (11%) [7], although this population underwent autologous IBR. Fat necrosis leads to volume loss and hardening of the reconstructed breast and particularly occurs when RT is given after IBR using free tissue transfer of skin and fat only (e.g. deep inferior epi-gastric perforator; DIEP flap). Therefore in such cases it would be preferable to delay autologous reconstruction until after the completion of RT. Other authors reported good outcome data after post-TRAM RT when delivery of RT was optimised [8]. There are however no randomised studies on the effect of RT on autologous reconstructions and most studies are heteregenous, uncontrolled and retrospective in nature [9].

The other concern regarding RT in the reconstructed breast is related to the use of implants, either alone or in conjunction with a flap reconstruction. The fibrosis in particular often causes subsequent shrinkage of the reconstructed breast around the implant – termed 'capsular contraction'. One study compared 39 irradiated implant reconstructed breasts with 338 non-irradiated reconstructions and found a significant negative effect on the reconstructive outcome with implants [10], the main complications being capsular contracture and post-operative pain. 43% of patients underwent a subsequent capsulotomy. Capsular contraction results in poor aesthetic outcomes in many cases, sometimes even requiring further flap surgery. This has led some surgeons to recommend that IBR using implants, including SSM, be avoided if it is known that a patient is likely to require postoperative RT [6]. However, more recently, in a prospective analysis evaluating unilateral postoperative chest wall RT in bilateral tissue expander/implant reconstruction patients, the authors observed that for the most patients, the degree of capsular contracture was higher on the irradiated side, yet overall symmetry, aesthetic results, and patient satisfaction remained high. Only in 10 percent of patients, contracture of the irradiated breast was two modified Baker grades. These observations support the notion that immediate breast reconstruction using tissue expansion and implants is an acceptable option for women undergoing mastectomy for breast cancer [11]. Moreover capsule formation can be treated with capsulotomy or capsulectomy. Despite the higher incidence of complications associated with PMRT, most patients remain satisfied with IBR [12]. In a retrospective study [12], 68 IBRs who received postoperative RT were compared with 75 IBRs who did not. It was found that although capsular contracture rates were higher in the irradiated group (68% v 40%, respectively), acceptable cosmetic outcome was achieved in most patients (good or better in 80% v 88%, p-value: non-significant). Patient satisfaction was significantly higher among non-irradiated patients (67% v 88%), and 72% of those irradiated said that they would still chose the same form of reconstruction again.

The adverse effects of RT on SSM and IBR can be minimised by optimising RT delivery and by placing a sub-pectoral saline-filled tissue expander in order to facilitate delayed revision of reconstruction using an implant, an autologous flap with or without implant after removing the tissue expander [13,14]. This new concept, known as immediate-delayed reconstruction, can avoid the aesthetic and radiation-delivery problems that can occur after IBR [13].

## SSM after RT

Hultman and Daiza investigated the effects of previous RT on subsequent SSM and IBR in 37 breasts, although not all patients had received previous RT [15]. TRAM and LD flaps and implant reconstructions were all included. 9 patients (24%) had a SSM flap complication of which 5 required re-operations. Previous irradiation and diabetes were found to be significant risk factors for complications. The issue of pre-operative RT and SSM was also investigated by Disa et al [16]. Their study included only 11 patients who underwent SSM and IBR after developing LRs following previous breast conservation surgery and RT. They observed that all the flaps survived, one patient developed partial thickness skin flap loss and two developed capsular contractures, leading them to conclude that SSM and IBR can be safely performed in previously irradiated breasts. Furthermore, Benediktsson and Perbeck have shown that RT does not significantly compromise the skin circulation of the breast [17].

More recently, Bacilious et al reported that tissue expansion was reliable with low morbidity in Hodgkin's patients with prior mantle irradiation [18]. Second-stage placement of permanent implants yielded good aesthetic results without significant capsular contracture or compromise of the prosthetic breast reconstruction.

However, these studies are small and larger studies with longer follow-up are required to verify these findings.

## Conclusion

We conclude that, as long as a higher complication rate is accepted and patients are fully informed, it would appear safe for women to undergo SSM and IBR after breast RT or before PMRT. Until better methods of RT delivery are developed to minimise complications, women at a high risk of requiring PMRT, can be safely offered SSM and IBR with a sub-pectoral saline-filled tissue expander and this can be replaced with a permanent prosthesis or converted into an autologous flap reconstruction after the completion of RT.

**Figure 1:**
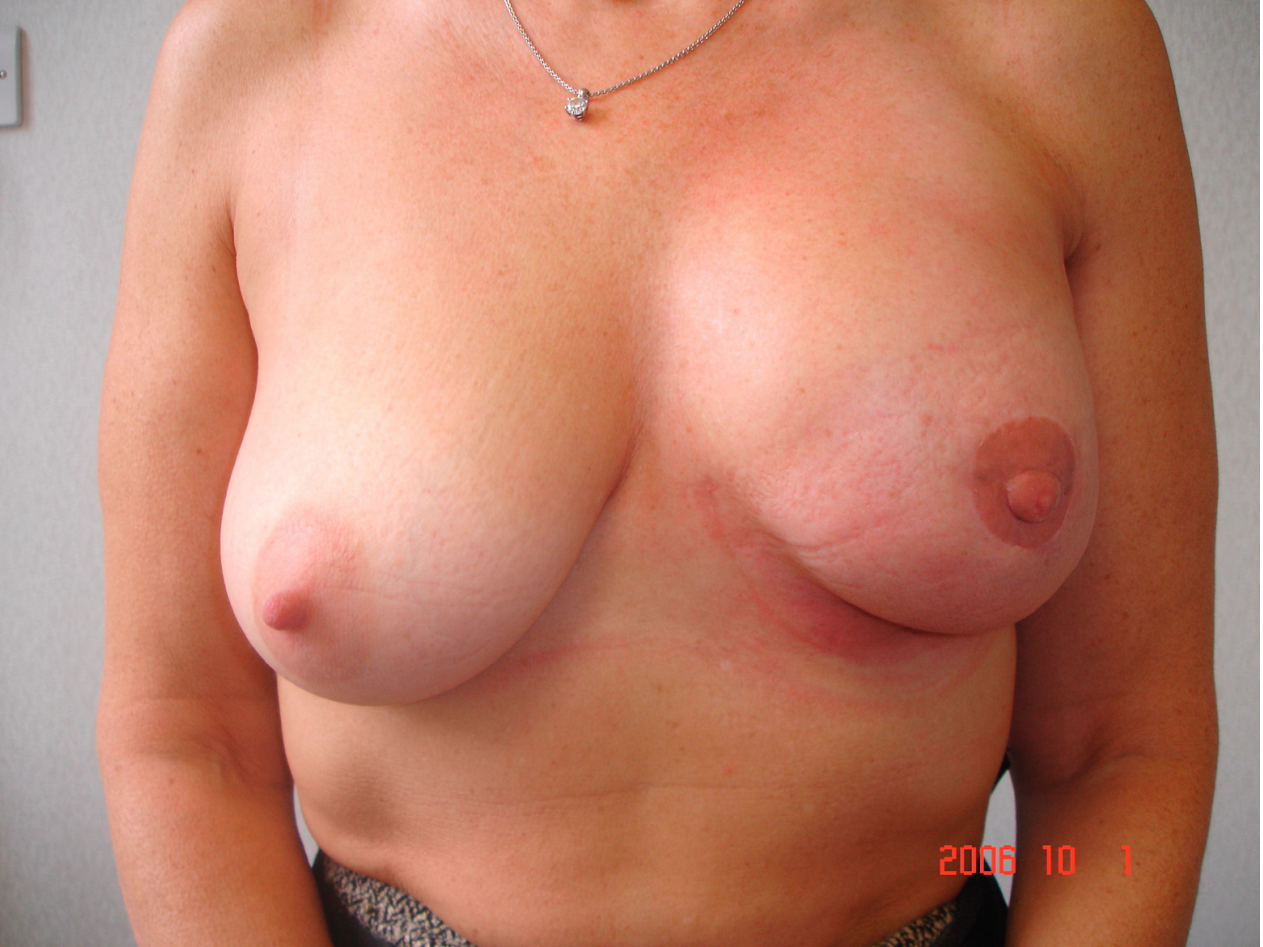
This 47 year old woman had left SSM and immediate latissimus dorsi (LD) flap reconstruction for node-positive breast cancer (T2N1M0). She subsequently had chemotherapy and RT. Thereafter, she had capsulotomy and nipple reconstruction using a local flap.
